# Case Report: Bow Hunter Syndrome—One Reason to Add Non-gravity Dependent Positional Nystagmus Testing to Your Clinical Neuro-Otologic Exam

**DOI:** 10.3389/fneur.2021.814998

**Published:** 2021-12-20

**Authors:** Michael C. Schubert, Nathaniel Carter, Sheng-fu Larry Lo

**Affiliations:** ^1^Laboratory of Vestibular NeuroAdaptation, Department of Otolaryngology-Head and Neck Surgery, Johns Hopkins University School of Medicine, Baltimore, MD, United States; ^2^Department of Physical Medicine and Rehabilitation, Johns Hopkins University School of Medicine, Baltimore, MD, United States; ^3^Maryland Center for Neuro-Ophthalmology & Neuro-Otology, Columbia, MD, United States; ^4^Department of Neurosurgery, Zucker School of Medicine at Hofstra/Northwell, Great Neck, NY, United States

**Keywords:** positional nystagmus, positional vertigo, Bow Hunter's syndrome, neurological examination, downbeat nystagmus

## Abstract

This case study describes transient downbeat nystagmus with vertigo due to a bilateral Bow Hunters Syndrome that was initially treated for 7 months as a peripheral benign paroxysmal positional vertigo. Normal static angiography and imaging studies (magnetic resonance, computed tomography) contributed to the mis-diagnosis. However, not until positional testing with the patient in upright (non-gravity dependent) was a transient downbeat nystagmus revealed with vertigo. The patient was referred for neurosurgical consult. Unfortunately, surgery was delayed due to suicidal ideation and hospitalization. Eventually, vertigo symptoms resolved following a C4-5 anterior cervical dissection and fusion. This case highlights the critical inclusion of non-gravity dependent position testing as an augment to the positional testing component of the clinical examination as well as the extreme duress that prolonged positional vertigo can cause.

## Introduction

Downbeat nystagmus induced from position change is a concerning clinical finding in patients with a history of positional vertigo. Although the posterior semicircular canal is more likely to be the culprit for positional vertigo when downbeat nystagmus (i.e., apogeotropic nystagmus due to inhibition of the cupula) is genuinely due to a peripheral vestibular cause in the head-hanging position (1–3), the clinician must remain suspicious of central nervous system pathology. The more common, non-peripheral vestibular causes for positional downbeat nystagmus comprise migraine (4) and cerebellar pathology including ectopia (5). Less common etiologies include spinocerebellar ataxia type 6 (6), multiple system atrophy (7), or superior cerebellar peduncle neoplasm (8). A rare, vascular cause for downbeat nystagmus with change in head position is bow hunter's syndrome (BHS), which can mimic a vestibular pathophysiology (9). Vertigo and dizziness, respectively, are the second and third most commonly reported symptom (syncope being the 1st) in BHS (10). BHS refers to the anatomical positioning of an archer/hunter pulling back on the bow, which involves lateral flexion of the cervical spine with a concurrent abduction of the ipsilateral shoulder. First described in 1978 after a patient suffered a lateral medullary stroke (11), signs and symptoms occur due to vascular compression (dynamic stenosis) of the vertebral arteries (12). For an excellent review on BHS please see Davis and Kane (13).

## Case Description

### History

A 56 year-old male reported symptoms of vertigo, imbalance, and brief “buzzing in both ears,” prior to having a “black out” sensation whenever he turned his head to the right. Turning the head back to a straight position restored the vision symptoms. He reported having fallen because of his vision blacking out and vertigo sensations. He is a mechanic and smokes 1.5 packs/day of cigarettes. He has a history of head trauma 30 years ago, Grave's disease s/p radioactive Iodine therapy, and hypertension. There was no prior surgical history. He has no history of migraine nor any sensitivity to changes in barometric pressure or visual motion. In the past (prior to vertigo onset), he described vomiting without dizziness after eating, which was ultimately related to a gastric ulcer. His prior workup included a normal otolaryngology examination, audiogram and mild chronic microvascular ischemic changes of the brain magnetic resonance (MR). MR angiogram of the brain was normal. MR angiogram of the neck revealed right greater then left plaque formation involving the proximal portions of the internal carotid arteries with no definite significant stenosis. Static computed tomography angiogram (CTA) of the head was normal. Computerized tomography (CT) angiogram of the neck revealed 50% narrowing of the right and 30% narrowing of the left proximal portion of the internal carotid arteries. CT of the temporal bones was normal. The patient was diagnosed with benign paroxysmal positional vertigo (BPPV) affecting his right anterior semicircular canal (aSCC) and over a period of 7 months was treated with the Demi-Semont, Semont for the right aSCC, and Brandt-Daroff exercises (14–16), without remission of symptoms and incongruent with current clinical practice guidelines (17). After having tried various positional maneuvers, the patient reported a less intense vertigo that was occurring more frequently (Table 1). However, he resorted to sleeping upright in a recliner to avoid the vertigo while lying flat and was unable to work.

**Table 1:** Timeline illustrating the relevant data from the episode of care.

	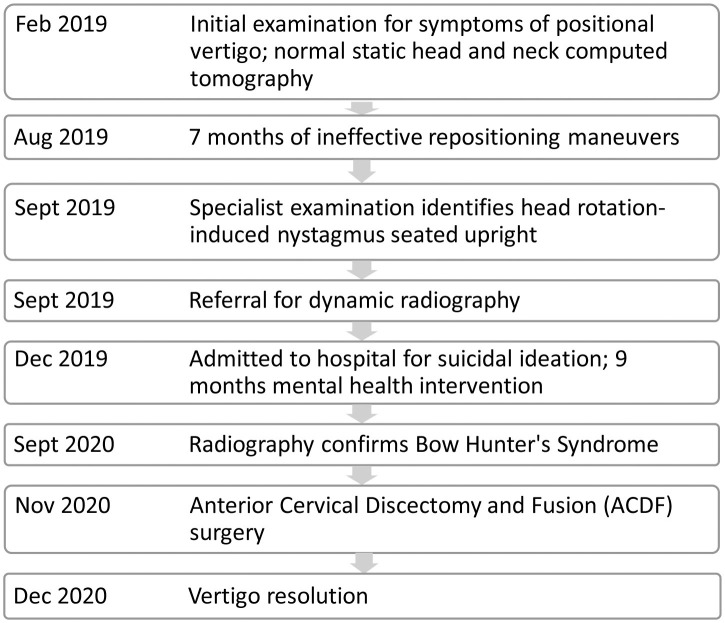	

### Diagnostic Assessment

#### Laboratory Exam

**Figure 1:**
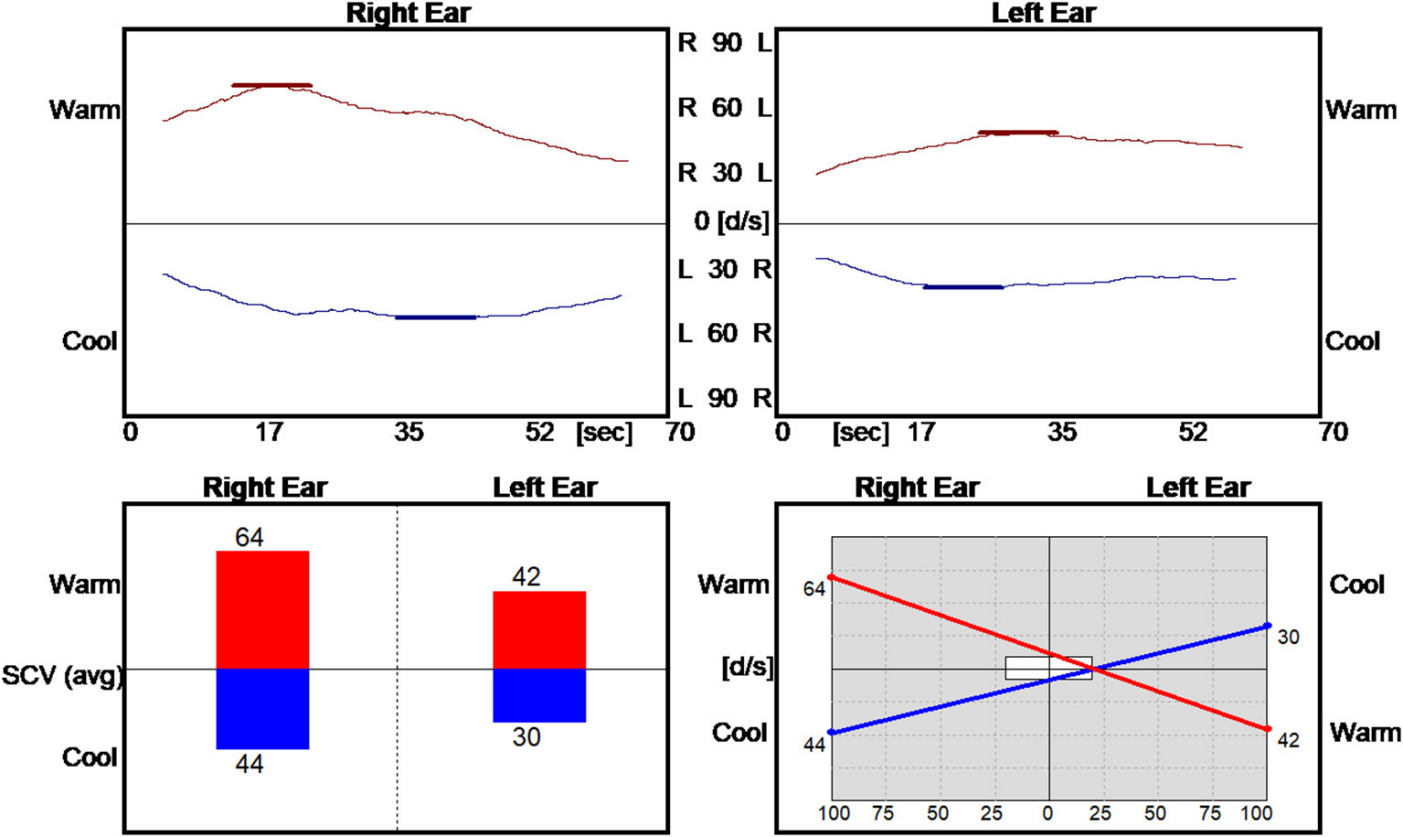
Normal caloric asymmetry but abnormally high total for the slow component eye velocity. SCV, slow component velocity; d/s, degrees/second. The caloric asymmetry is 20% (Table 1). R, right; L, left.

The video-nystagmography record of smooth pursuit and saccade testing were normal. The vestibulo-ocular reflex during active and passive sinusoid head rotation from 1 to 4 Hz was normal. The bi-thermal water caloric exam revealed a sum slow eye velocity of 180 d/s (Figure 1), exceeding the normal 45–157 d/s range of total slow eye velocities (18). He had a no asymmetry in the responses (≤ 20%).

#### Clinical Exam

**Figure 2:**
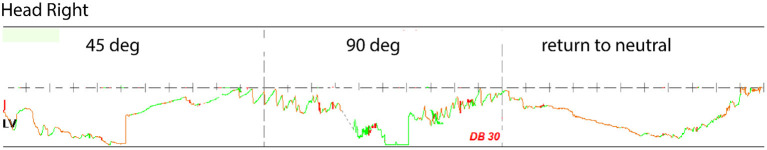
Downbeat nystagmus during positional testing and head rotated right. At 90 deg, the downbeat nystagmus has a peak velocity of 30 deg/sec, originally interpreted as evidence for otoconia dislodged within the lumen of the right anterior semicircular canal. LV, left vertical channel.

The patient presented in the clinic with a cautious gait and reduced gait speed. In room light, he had no spontaneous or gaze holding nystagmus. He did not show ocular misalignment (no tropia/phoria). Extraocular movements were intact for all directions. He could generate normal smooth pursuit and saccades for both horizontal and vertical direction. His vestibulo-ocular reflex was normal to rapid impulses in the planes of the right and left horizontal semicircular canals. Under video infrared exam, he had no spontaneous or gaze holding nystagmus. He developed a mild downbeat nystagmus after head shaking but had no mastoid vibration-induced nystagmus. He had no nystagmus from pinched-nose or glottis-closed Valsalva maneuvers. In the left Dix Hallpike position, he developed a sustained upbeat nystagmus with a mild dizziness sensation but did not report frank vertigo. In the right Dix Hallpike position, he developed a mild right beat nystagmus without dizziness or vertigo. The supine roll test was normal at 45 degrees right or left, however, once he rotated his head beyond 75 degrees to the right he developed downbeat nystagmus with vertigo and an impending sense of syncope (Figure 2). When seated upright, rightward head rotation of 90 degrees reproduced downbeat nystagmus with vertigo. If he added right shoulder abduction to the 90 deg rightward head rotation, the downbeat velocity of his nystagmus increased. He was referred to neuro-radiology and surgical consult.

#### Suicidal Ideation

While attempting to schedule the dynamic radiography, the patient began expressing feelings of depression and commented that he “wanted to blow his brains out.” During this interaction, the patient confirmed he had access to many guns. The scheduler was able to keep the patient occupied on the phone while another provider contacted emergency services. The patient was admitted 4 days to the hospital for suicidal ideation before discharge. Radiography was rescheduled for the next month. Unfortunately, he grew anxious awaiting the impending exam and a relative called to report the patient had been drinking heavily and was “non-functioning.” The relative reported he had recently been arrested twice within a short time of each other for driving while intoxicated. Radiography was delayed another 9 months during which time he sought mental health intervention.

#### Dynamic Radiographic Exam

**Figure 3:**
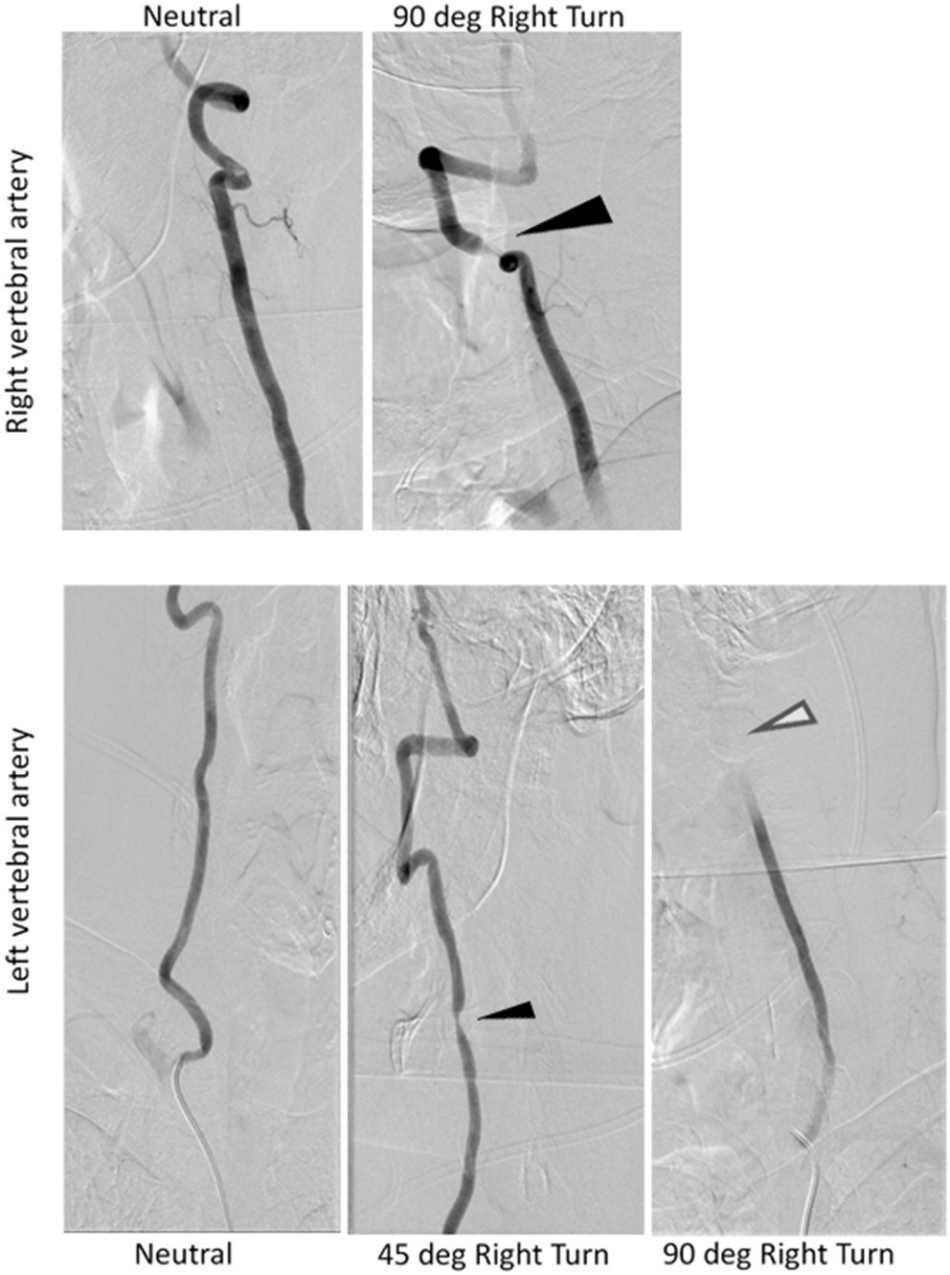
Radiograph evidence confirming severe stenosis of right and left vertebral arteries during rightward head rotation. Dark arrows reflect partial stenosis, white arrow reflects absent perfusion from complete stenosis.

Dynamic angiogram using digital subtraction angiography (DSA) revealed stenosis of the right vertebral artery at the C4-C5 level that increased with increasing amplitude of head rotation to the right (Figure 3). He also had a position-dependent severe stenosis of the left vertebral artery with partial to complete occlusion with increasing amplitude of right head rotation. Osteophytes were present at the C4-5 level on the right side deemed a ‘critical stenosis'. In addition, the left vertebral artery cinched off at the transition between V2-3 at the base of the C2 lateral mass where the vertebral artery turns lateral. A repeat CTA of the head was ordered to examine the cervical anatomy in consideration of surgical planning. The CTA revealed a severe stenosis of the V2 segment of the right vertebral artery at C4-5 secondary to uncovertebral joint arthropathy with osseous compression of the artery with right head rotation. This corresponds to degenerative changes worse at C4-5 and findings between neutral and rotational CT suggesting rotational instability. In addition, he had anterior subluxation of C1 on C2 with a marked stenosis of the left vertebral artery at C2. The subluxation stretches the vertebral artery during rightward rotation. He has no stenosis in the neutral position. There was ~45 and 60% stenosis of the right and left carotid bulbs due to atherosclerotic disease. He was diagnosed with Bow Hunter's syndrome due to dynamic stenosis of the left vertebral artery at C1-2 as well as stenosis of the right vertebral artery at C4-5 with a right-sided head turn.

#### Surgical Intervention and Follow Up

The patient's case was discussed at a multi-disciplinary neurovascular conference and it was postulated that simultaneous compression of the V2 segment of the right vertebral artery at C4-5 and V3 segment of the left vertebral artery at C2 with rightward rotation was causing his symptoms and that unilateral compression alone would be well-tolerated. The surgical plan thus formulated to perform a C4-5 anterior cervical discectomy and fusion (ACDF) to remove the dynamic compression of the right vertebral artery in order to see if that alone was sufficient to address his symptoms. The plan was to monitor him overnight after surgery to evaluate for symptom resolution and if not, the surgical team would move forward the next day with a posterior C1-2 fusion to address the dynamic compression on the left vertebral artery. The C4-5 ACDF was chosen as the initial approach due to its less morbid profile in terms of reduction of neck range of motion compared to a posterior C1-2 fusion. He underwent the procedure uneventfully and had immediate resolution of his symptoms during rightward head rotation (after surgery and in recovery). Given how well he was doing, he elected to be discharged home the same day. On his 6 week post-operative visit, he remained symptom free on neurological testing. Repeat video-ENG recording 6 months post-operative were normal for saccades, pursuit, and positional nystagmus tests. He had no vertigo or vision blackout during positional testing.

## Discussion

Our case is unique as it represents a positional nystagmus present in both gravity (head down) and non-gravity dependent (head up) positions due to BHS. Furthermore, our case is rare given both vertebral arteries were compromised, reported to occur in <14% of BHS (10). Recall, the patient did have both a horizontal or an upbeat positional nystagmus that occurred in head down position testing, which can be confusing to clinicians that may consider this to be of a peripheral vestibular origin. However, neither of these directions of nystagmus were concurrent with a sensation of vertigo. Instead, only the down beat nystagmus in the head hanging position was associated with vertigo as was the impending sense of syncope. Thus, our case highlights the importance of associating a patient's symptoms and history with the results of positional testing for nystagmus in both gravity and non-gravity dependent positions. Clinicians do not typically perform positional testing for nystagmus with the patients head in upright, though recent literature suggests using an upright head position to test for nystagmus can be useful to discern peripheral vestibular from vascular causes of vertigo (19–21).

In this case, dynamic angiogram using digital subtraction angiography (DSA) along with dynamic CT angiogram and carotid ultrasound with head rotations were critical to establish the diagnosis of bilateral BHS, but careful clinical examination first identified the atypical presentation of positional nystagmus.

Our case of BHS differs from others due to the predominant feature of down beating nystagmus. Recently, BHS was reported to cause nystagmus that varied in direction more than our case, also in non-gravity dependent head positions. Nomura et al. (21) described a patient developing left beat nystagmus due to head rotation to the left while seated upright. When the patient's head was turned back to the center there was a transitional down beating nystagmus followed by right beating nystagmus. The authors postulated that the transient down beating nystagmus resulted from transient ischemia at the brainstem, which resolved once hypertension was properly managed. Another unique feature of this case was that static CTA did not reveal overt stenosis or hypoplasia of the right V2 segment or the left V3 segment of the vertebral artery. Rather than static unilateral compression of the vertebral artery, here we have a case of bilateral dynamic compression of the vertebral artery that lead the patient to become symptomatic on rightward head rotation. As such, our hypothesis was that decompression and stabilization across the less operatively morbid side alone (right C4-5) would be sufficient to address the patient's symptomatology. This case highlights the need for heightened vigilance for the possibility of dynamic vertebral artery compression as the cause of BHS and dynamic imaging such as a dynamic CTA or DSA should be pursued in the appropriate clinical setting. Lida et al. (9) reported a case of downbeat nystagmus caused by compression of the non-dominant vertebral artery, though did not perform positional testing with the head in a position other than upright.

One notable feature of our case presentation was the consistent onset of tinnitus (“buzzing in both ears“) seconds before the report of the positional vertigo. Although the occurrence of tinnitus with BPPV is not very common, it does occur. In a series of 171 patients with BPPV, 19.3% reported the presence of tinnitus concurrently with the onset of vertigo (22). To the best of our knowledge, the reported incidence of tinnitus in BHS seems to be <10% (10). Our case and other literature therefore seems to suggest that patients with tinnitus and vertigo both triggered by positional testing should raise suspicion for a vascular cause of symptoms, including BHS.

A final, frightening novelty of this case is the suicidal ideation mandating hospitalization thus highlighting how the symptom of vertigo significant impairs quality of life. Health utility indexes (HUI) reflect the perception of health by considering the level of physical, mental, and social functioning as associated with a condition. Scores range from 0 (death) and 1.00 (optimal health). Negative scores are possible and imply a state of health worse than death. Patients with vestibular conditions are known to report HUI scores lower than healthy controls. For example, HUI scores for superior semicircular canal dehiscent syndrome are reported to be 0.65, similar with unilateral vestibular hypofunction (0.63), but much higher than HUI scores reported by those with bilateral vestibular hypofunction (0.39) (23, 24). As comparison, patient with diabetes report HUI scores ~0.8 (25).

## Conclusion

Clinicians managing patients with reports of vertigo should proceed with caution when downbeat nystagmus presents during positional testing. While repositioning maneuvers for BPPV typically have a high success rate, careful attention to the patient history and inclusion of upright testing for nystagmus may be essential for the early indication of a possible vascular etiology. Adding these components to the initial examination of vertigo may have precluded our patient from receiving repeated repositioning maneuvers and experiencing suicidal ideation.

## Data Availability Statement

The original contributions presented in the study are included in the article/supplementary material, further inquiries can be directed to the corresponding author/s.

## Ethics Statement

Written informed consent was obtained from the individual(s) for the publication of any potentially identifiable images or data included in this article.

## Author Contributions

All authors listed have made a substantial, direct, and intellectual contribution to the work and approved it for publication.

## Conflict of Interest

The authors declare that the research was conducted in the absence of any commercial or financial relationships that could be construed as a potential conflict of interest.

## Publisher's Note

All claims expressed in this article are solely those of the authors and do not necessarily represent those of their affiliated organizations, or those of the publisher, the editors and the reviewers. Any product that may be evaluated in this article, or claim that may be made by its manufacturer, is not guaranteed or endorsed by the publisher.

## References

[1] Büki B Simon L Garab S Lundberg YW Jünger H Straumann D. Sitting-up vertigo and trunk retropulsion in patients with benign positional vertigo but without positional nystagmus. J Neurol Neurosurg Psychiatry. (2011) 82:98–104. 10.1136/jnnp.2009.199208 20660923 PMC4196320

[2] Califano L Salafia F Mazzone S Melillo MG Califano M. Anterior canal BPPV and apogeotropic posterior canal BPPV: two rare forms of vertical canalolithiasis. Acta Otorhinolaryngol Ital. (2014) 34:189–97. 24882928 PMC4035840

[3] Helminski JO. Peripheral downbeat positional nystagmus: apogeotropic posterior canal or anterior canal BPPV. J Neurol Phys Ther. (2019) 43(Suppl. 2):S8–13. 10.1097/NPT.0000000000000267 30883487

[4] Young AS Lechner C Bradshaw AP MacDougall HG Black DA Halmagyi GM. Capturing acute vertigo: a vestibular event monitor. Neurology. (2019) 92:e2743–53. 10.1212/WNL.0000000000007644 31092626

[5] Bertholon P Bronstein AM Davies RA Rudge P Thilo KV. Positional down beating nystagmus in 50 patients: cerebellar disorders and possible anterior semicircular canalithiasis. J Neurol Neurosurg Psychiatry. (2002) 72:366–72. 10.1136/jnnp.72.3.366 11861698 PMC1737794

[6] Yabe I Sasaki H Takeichi N Takei A Hamada T Fukushima K. Positional vertigo and macroscopic downbeat positioning nystagmus in spinocerebellar ataxia type 6 (SCA6). J Neurol. (2003) 250:440–3. 10.1007/s00415-003-1020-5 12700909

[7] Lee JY Lee WW Kim JS Kim HJ Kim JK Jeon BS. Perverted head-shaking and positional downbeat nystagmus in patients with multiple system atrophy. Mov Disord. (2009) 24:1290–5. 10.1002/mds.22559 19412932

[8] Lea J Lechner C Halmagyi GM Welgampola MS. Not so benign positional vertigo: paroxysmal downbeat nystagmus from a superior cerebellar peduncle neoplasm. Otol Neurotol. (2014) 35:e204–5. 10.1097/MAO.0000000000000245 24643027

[9] Lida Y Murata H Johkura K Higashida T Tanaka T Tateishi K. Bow Hunter's syndrome by nondominant vertebral artery compression: a case report, literature review, and significance of downbeat nystagmus as the diagnostic clue. World Neurosurg. (2018) 111:367–72. 10.1016/j.wneu.2017.12.167 29309982

[10] Jost GF Dailey AT. Bow hunter's syndrome revisited: 2 new cases and literature review of 124 cases. Neurosurg Focus FOC. (2015) 38:E7. 10.3171/2015.1.FOCUS14791 25828501

[11] Sorensen BF. Bow hunter's stroke. Neurosurgery. (1978) 2:259–61. 10.1227/00006123-197805000-00013 732978

[12] Fox MW Piepgras DG Bartleson JD. Anterolateral decompression of the atlantoaxial vertebral artery for symptomatic positional occlusion of the vertebral artery. Case report. J Neurosurg. (1995) 83:737–40. 10.3171/jns.1995.83.4.0737 7674027

[13] Davis DD Kane SM. Rotation Vertebral Artery Syndrome. Treasure Island, FL: StatPearls Publishing (2020). Available online at: https://www.ncbi.nlm.nih.gov/journals/NBK559022/32644448

[14] Brandt T Daroff RB. Physical therapy for benign paroxysmal positional vertigo. Arch Otolaryngol Head Neck Surg. (1980) 106:484–5. 10.1001/archotol.1980.00790320036009 7396795

[15] Honrubia V Baloh RW Harris MR Jacobson KM. Paroxysmal positional vertigo syndrome. Am J. Otol. (1999) 20:465–70, 10. 10431888

[16] Vannucchi P Pecci R Giannoni B Di Giustino F Santimone R Mengucci A. Canal benign paroxysmal positional vertigo: some clinical and therapeutic considerations. Audiol Res. (2015) 5:130. 10.4081/audiores.2015.130 26557364 PMC4627115

[17] Bhattacharyya N Gubbels SP Schwartz SR Edlow JA El-Kashlan H Fife T. Clinical practice guideline: benign paroxysmal positional vertigo (update) executive summary. Otolaryngol Head Neck Surg. (2017) 156:403–16. 10.1177/0194599816689660 28248602

[18] Huh YE Kim JS Kim HJ Park SH Jeon BS Kim JM. Vestibular performance during high-acceleration stimuli correlates with clinical decline in SCA6. Cerebellum. (2015) 14:284–91. 10.1007/s12311-015-0650-3 25624155

[19] Martellucci S Malara P Castellucci A. Upright BPPV protocol: feasibility of a new diagnostic paradigm for lateral semicircular canal benign paroxysmal positional vertigo compared to standard diagnostic maneuvers. Front Neurol. (2020) 11:578305. 10.3389/fneur.2020.578305 33329319 PMC7711159

[20] Malara P Castellucci A Martellucci S. Upright head roll test: a new contribution for the diagnosis of lateral semicircular canal benign paroxysmal positional vertigo. Audiol Res. (2020) 10:236. 10.4081/audiores.2020.236 32676175 PMC7358984

[21] Nomura Y Toi T Ogawa Y Oshima T Saito Y. Transitional nystagmus in a Bow Hunter's Syndrome case report. BMC Neurol. (2020) 20:435. 10.1186/s12883-020-02009-3 33256636 PMC7706255

[22] Barozzi S Socci M Ginocchio D Filipponi E Martinazzoli MG Cesarani A. Benign paroxysmal positional vertigo and tinnitus. Int Tinnitus J. (2013) 18:16–9. 10.5935/0946-5448.20130003 24995895

[23] Ocak I Topsakal V Van de Heyning P Van Haesendonck G Jorissen C van de Berg R. Impact of superior Canal Dehiscence syndrome on health utility values: a prospective case-control study. Front Neurol. (2020) 11:552495. 10.3389/fneur.2020.552495 33133004 PMC7578361

[24] Sun DQ Ward BK Semenov YR Carey JP Della Santina CC. Bilateral vestibular deficiency: quality of life and economic implications. JAMA Otolaryngol Head Neck Surg. (2014) 140:527–34. 10.1001/jamaoto.2014.490 24763518 PMC4208975

[25] Zhang P Brown MB Bilik D Ackermann RT Li R Herman WH. . Diabetes Care. (2012) 35:2250–6. 10.2337/dc11-2478 22837369 PMC3476906

